# Leveraging Clinical Informatics Tools to Extract Cumulative Anthracycline Exposure, Measure Cardiovascular Outcomes, and Assess Guideline Adherence for Children With Cancer

**DOI:** 10.1200/CCI.21.00099

**Published:** 2021-10-29

**Authors:** David H. Noyd, Amy Berkman, Claire Howell, Steve Power, Susan G. Kreissman, Andrew P. Landstrom, Michel Khouri, Kevin C. Oeffinger, Warren A. Kibbe

**Affiliations:** ^1^ Department of Pediatrics, The University of Oklahoma Health Sciences Center, Oklahoma City, OK; ^2^ Department of Pediatrics, Duke University Medical Center, Durham, NC; ^3^ Duke Cancer Institute, Durham, NC; ^4^ Division of Cardiology and Department of Cell Biology, Department of Pediatrics, Duke University Medical Center, Durham, NC; ^5^ Department of Medicine, Duke University Medical Center, Durham, NC; ^6^ Department of Biostatistics and Bioinformatics, Duke University, Durham, NC

## Abstract

Cardiovascular disease is a significant cause of late morbidity and mortality in survivors of childhood cancer. Clinical informatics tools could enhance provider adherence to echocardiogram guidelines for early detection of late-onset cardiomyopathy.

**METHODS:**

Cancer registry data were linked to electronic health record data. Structured query language facilitated the construction of anthracycline-exposed cohorts at a single institution. Primary outcomes included the data quality from automatic anthracycline extraction, sensitivity of International Classification of Disease coding for heart failure, and adherence to echocardiogram guideline recommendations.

**RESULTS:**

The final analytic cohort included 385 pediatric oncology patients diagnosed between July 1, 2013, and December 31, 2018, among whom 194 were classified as no anthracycline exposure, 143 had low anthracycline exposure (< 250 mg/m^2^), and 48 had high anthracycline exposure (≥ 250 mg/m^2^). Manual review of anthracycline exposure was highly concordant (95%) with the automatic extraction. Among the unexposed group, 15% had an anthracycline administered at an outside institution not captured by standard query language coding. Manual review of echocardiogram parameters and clinic notes yielded a sensitivity of 75%, specificity of 98%, and positive predictive value of 68% for International Classification of Disease coding of heart failure. For patients with anthracycline exposure, 78.5% (n = 62) were adherent to guideline recommendations for echocardiogram surveillance. There were significant association with provider adherence and race and ethnicity (*P* = .047), and 50% of patients with Spanish as their primary language were adherent compared with 90% of patients with English as their primary language (*P* = .003).

**CONCLUSION:**

Extraction of treatment exposures from the electronic health record through clinical informatics and integration with cancer registry data represents a feasible approach to assess cardiovascular disease outcomes and adherence to guideline recommendations for survivors.

## INTRODUCTION

Cardiovascular disease (CVD) is a significant cause of late morbidity and mortality in survivors of childhood cancer.^1^ An analysis from the Childhood Cancer Survivor Study (CCSS) showed an increased incidence of serious CVD, including cardiomyopathy, coronary artery disease, arrhythmias, and valvular disease, among survivors of childhood cancer compared with siblings.^2^ Indeed, survivors demonstrated seven times higher mortality rates compared with the general population, with cardiac causes of death second only to subsequent malignancy.^1-3^ The cumulative incidence of cardiomyopathy by age 45 years was 4.8% among all survivors with an earlier onset in adulthood compared with siblings and higher incidence rates for survivors with anthracycline exposure.^2,4^

**CONTEXT**

**Key Objective**
To automatically extract cumulative anthracycline exposure from the electronic health record through standard query language, construct cardiovascular risk-based cohorts of children with cancer, and assess provider adherence to guideline recommendations for echocardiograms.
**Knowledge Generated**
Automatic extraction of cumulative anthracycline exposure was highly consistent with manual chart review for children with cancer, which represents a feasible approach for cardiovascular risk stratification of survivors. Significant differences in provider adherence to echocardiogram guidelines were observed with regard to patient race and ethnicity and primary language.
**Relevance**
Anthracycline exposure is an important predictor of late cardiotoxicity, and automatic extraction of this key data element from the electronic health record, integrated with cancer registry data, can guide equitable population health management of survivors of childhood cancer. Furthermore, this serves as a model to promote interoperability at other institutions and expansion to include other critical treatment exposures.

Chemotherapy, most notably anthracyclines, and chest radiation are cardiotoxic.^5^ There is a strong dose-response relationship between cumulative anthracycline dose and cardiomyocyte injury, primarily with a marked increase in risk after 250 mg/m^2^.^6^ Although recent cardioprotective strategies, such as dexrazoxane,^7,8^ and reduction of anthracycline exposures resulted in a modest decrease in the cumulative incidence of heart failure in survivors treated in more recent decades,^9^ cumulative anthracycline dose persists as a major predictor of CVD. Socioeconomic factors influence overall cardiovascular health^10^ and survivors report lower physical activity levels,^11^ which could further exacerbate treatment-related effects.^12^ Therefore, early diagnosis of treatment-related cardiovascular sequelae represents key targets for interventions aimed at cardioprotection, such as adherence to screening guidelines, preventive care, and healthy lifestyles.

As the knowledge of late cardiac effects expands through well-designed longitudinal cohort studies, such as the CCSS, the implementation of guideline-based care is essential. On the basis of treatment exposures, the Children's Oncology Group (COG) routinely publishes guidelines for long-term follow-up care, including detection of cardiotoxicity.^13^ The International Guideline Harmonization Group provides another layer for evidence-based survivorship care.^14^ Together these inform screening guidelines for early detection of cardiovascular complications.^15^ Analyses have proven the cost-effectiveness of following guideline-based screening frequency of echocardiograms.^16,17^ Moreover, gene-wide association studies and bioinformatics advances in precision medicine promise to further refine risk stratification for anthracycline-induced cardiotoxicity among survivors.^18-22^

Learning health systems^23,24^ and the application of real-world data to improve survivorship care are synergistic with national efforts in clinical oncology such as the Cancer Moonshot to enhance data sharing.^25^ The Childhood Cancer Data Initiative aims to build an infrastructure to integrate data from multiple sources,^26^ and the Cancer Informatics for Cancer Centers organization reflects the growing interest on harness informatics, data science, and population science methods within the field of oncology.^27^ The Minimal Common Oncology Data Elements (mCODE) Initiative focuses on the application of data standards to promote interoperability of cancer-related information.^28^ The Childhood Cancer Data Initiative and mCODE need tangible, reproducible methods, such as the integration of electronic health record (EHR) and cancer registry data,^29^ to apply current knowledge to improve survivorship care and cultivate multi-institutional collaborations.

The primary aim of this study was to use a clinical informatics approach to automatically extract cumulative anthracycline exposure data from the EHR to construct cardiovascular risk-adapted cohorts of children with cancer to assess early CVD burden and adherence to guideline-based recommendations for echocardiogram surveillance.

## METHODS

### Cancer Registry and Patient Information

**FIG 1.**
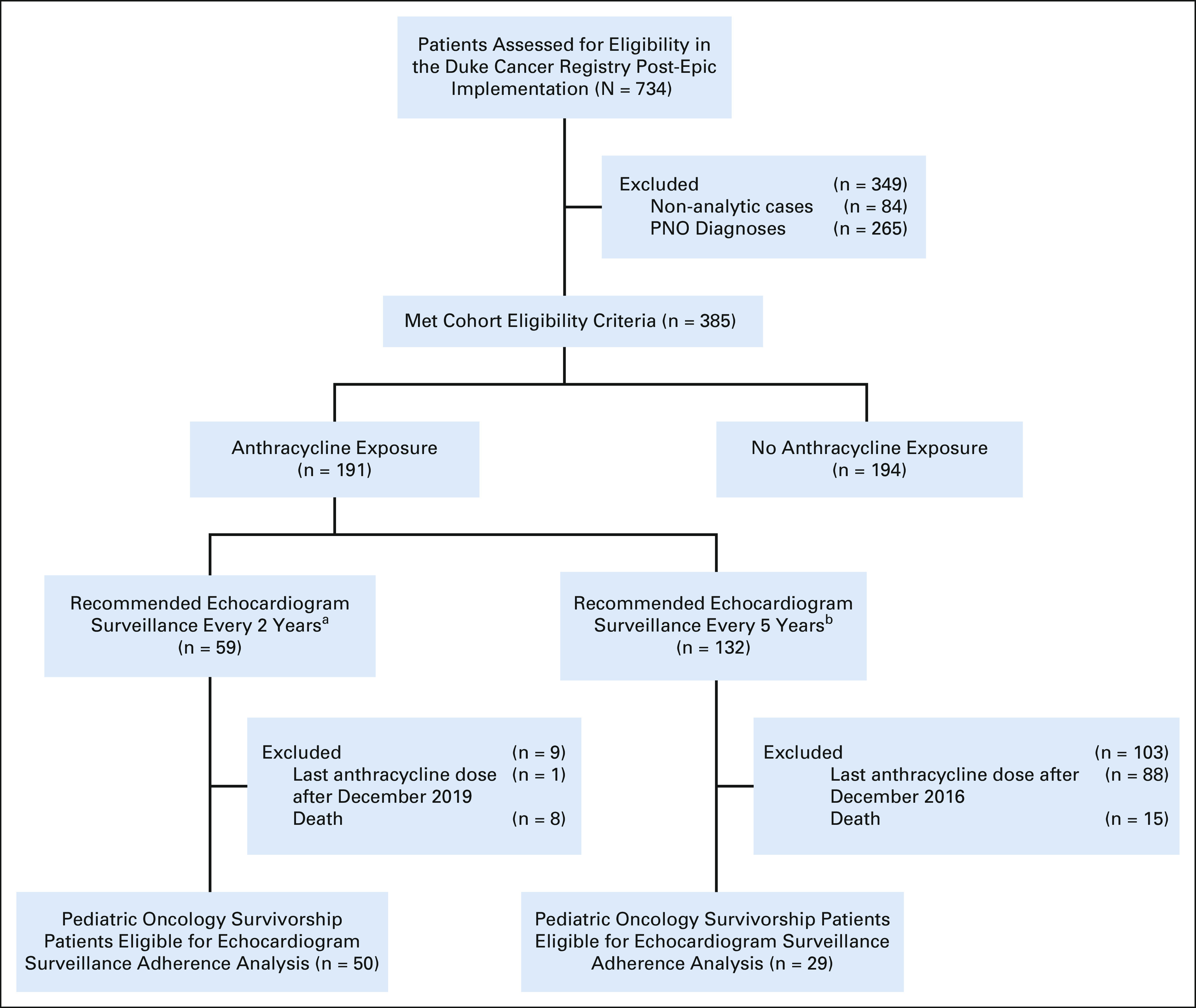
Construction of the final analytic childhood cancer cumulative anthracycline exposure cohort on the basis of inclusion and exclusion criteria from cancer registry data for patients diagnosed between July 1, 2013, and December 31, 2018. ^a^Cumulative anthracycline dose ≥ 250 mg/m^2^ or cumulative anthracycline dose < 250 and chest radiation ≥ 15 Gy. ^b^Cumulative anthracycline dose < 250 mg/m^2^ and chest radiation < 15 Gy. PNO, pediatric neuro-oncology.

The Duke Health Institutional Review Board approved this study. Cancer centers accredited by the Commission on Cancer are required to report all newly diagnosed cases to the National Cancer Database.^30^ Cancer registry data provided the base cohort, and EHR data elements were integrated and linked by the medical record number. Cardiovascular risk-adapted cohorts were constructs based on patients with documented anthracycline exposure, ≤ 18 years of age at time of diagnosis, and reported to the cancer registry with a malignancy between July 1, 2013 (the date of EHR implementation) and December 31, 2018 (Fig 1). Only analytic cases were included, which are defined by the National Cancer Database as cases newly diagnosed and received all or part of the first course of treatment at the reporting facility. Patient age at diagnosis, race and ethnicity, and mortality data were captured from the cancer registry. Patient gender was extracted from the EHR using the Duke Enterprise Data Unification Content Explorer (DEDUCE). DEDUCE is an institutional self-service query tool that uses a data dictionary and standard query language (SQL) to automatically extract data elements from the Duke Medicine enterprise data warehouse.^31^ SQL was used to extract primary language data directly from the EHR database Clarity.

This research was submitted to and approved by the Duke University Health System Institutional Review Board (Pro00104185) on December 11, 2019.

### Disease Classification

The International Classification of Diseases for Oncology, Third Revision, from the cancer registry, and the disease groupings from the International Classification of Childhood Cancer, Third Revision,^32^ were used to identify a pediatric oncology cohort. Patients with a primary CNS tumor were excluded (n = 262), as they are historically less likely to receive anthracyclines.^5^ For cardiovascular outcomes, all International Classification of Diseases, Ninth Revision, Clinical Modification (ICD-9-CM) and International Classification of Diseases, Tenth Revision, Clinical Modification (ICD-10-CM) were extracted from the EHR using DEDUCE. Established classifications for cardiomyopathy, heart failure, atrial fibrillation, and ventricular arrhythmias provided a framework to capture major cardiac outcomes during the treatment and early survivorship periods (Appendix Table A1).^33-36^

### Anthracycline Exposure

Discrete data elements for cumulative anthracycline exposure were extracted through SQL coding from the Epic EHR reporting database Clarity. The lifetime dose-tracking table, built into Epic by the informatics team at Duke, captures the total cumulative dose for each type of anthracycline, measured in milligrams per meter squared. The body surface area was measured using the documented height and weight at the time of administration. Cumulative anthracycline exposure was calculated by the doxorubicin isotoxic equivalent dose with the total dose of doxorubicin multiplied by one, the total dose of daunorubicin multiplied by 0.5, the total dose of epirubicin multiplied by 0.67, the total dose of idarubicin multiplied by 5, and the total dose of mitoxantrone multiplied by 4.^13,37^ High and low anthracycline exposure cutoffs were defined as a cumulative dose of ≥ 250 mg/m^2^ and < 250 mg/m^2^, respectively (Appendix Tables A2 and A3).

### Echocardiogram Guideline Adherence and Cohort Construction

DEDUCE was used to automatically extract the date of every echocardiogram from the EHR on the basis of Current Procedural Terminology (CPT) coding.^38^ Congruent with the COG Long-Term Follow-Up Guidelines, Version 5.0,^13^ adherence was defined as an echocardiogram within 27 months of the last administration of an anthracycline for the patients with cumulative exposure ≥ 250 mg/m^2^ or exposure < 250 mg/m^2^ and chest radiation ≥ 15 Gy. For patients with cumulative exposure < 250 mg/m^2^ and chest radiation < 15 Gy, adherence was defined as an echocardiogram within 63 months of the last administration of an anthracycline.

### Manual EHR Review

Manual EHR review of patient data including cumulative anthracycline exposure, chest radiation data (which were not captured in the SQL methods), causes of death, and echocardiogram parameters for heart failure facilitated both quality assurance and a sensitivity analysis for International Classification of Disease (ICD) coding of heart failure in this cohort. Study data were collected and managed using Research Electronic Data Capture (REDCap) tools hosted at Duke University Medical Center.^39,40^ REDCap is a secure, web-based software platform designed to support data capture for research studies, providing (1) an intuitive interface for validated data capture, (2) audit trails for tracking data manipulation and export procedures, (3) automated export procedures for seamless data downloads to common statistical packages, and (4) procedures for data integration and interoperability with external sources. The REDCap project designer facilitated discrete fields for each patient to record the date of each echo, echocardiogram parameters (ejection fraction and shortening fraction), ICD cardiac codes, diagnosis of heart failure by cardiology, and documented heart failure symptoms.

### Statistical Analyses

Patients reported to the cancer registry with a non-CNS diagnosis were grouped by no, low, and high anthracycline exposures. Continuous variables are presented as means (standard error [SE]), and differences were compared using the analysis of variance test across the three groups. Categorical variables are presented as counts (proportions), and the χ^2^ test was used to compare differences. For patients with anthracycline exposure and who met the eligibility criteria for echocardiogram guideline adherence, differences in continuous variables were compared using the *t*-test, and differences in categorical variables were compared using the χ^2^ test. All statistical analyses were conducted using Stata/SE version 16.1 (Stata Corp LLC, College Station, TX).

## RESULTS

### Post-EHR Implementation Anthracycline Exposure Cohort Construction

A total of 734 patients age ≤ 18 years at the time of diagnosis were captured in the institutional cancer registry between July 1, 2013, and December 31, 2018. We excluded 265 CNS patients on the basis of International Classification of Diseases for Oncology, Third Revision coding, and International Classification of Childhood Cancer, Third Revision classification.^32^ Of the remaining 469 patients, 84 patients coded as nonanalytic cases in the cancer registry were excluded, leaving a total of 385 patients in the final analytic cohort (Fig 1).

SQL was used to automatically extract cumulative anthracycline exposure from Clarity. A total of 191 patients received an anthracycline, among whom 48 received a high cumulative dose (≥ 250 mg/m^2^) and 143 received an intermediate or low dose (< 250 mg/m^2^). One hundred ninety-four patients did not receive anthracyclines. The high anthracycline exposure group received a mean cumulative anthracycline dose of 362.1 (SE 8.2) mg/m^2^ compared with a mean dose of 121.1 (SE 4.9) mg/m^2^ in the low anthracycline exposure group and were more likely to receive dexrazoxane (58% *v* 6%) for cardioprotection (*P* < .001). There were no statistical differences between the high, low, and no anthracycline-exposed groups with regard to sex (*P* = .691) or race and ethnicity (*P* = .783). There was significant heterogeneity in the age at diagnosis (*P* < .001) and diagnosis of Trisomy 21 (*P* = .043) among the different groups (Table 1).

**TABLE 1.**
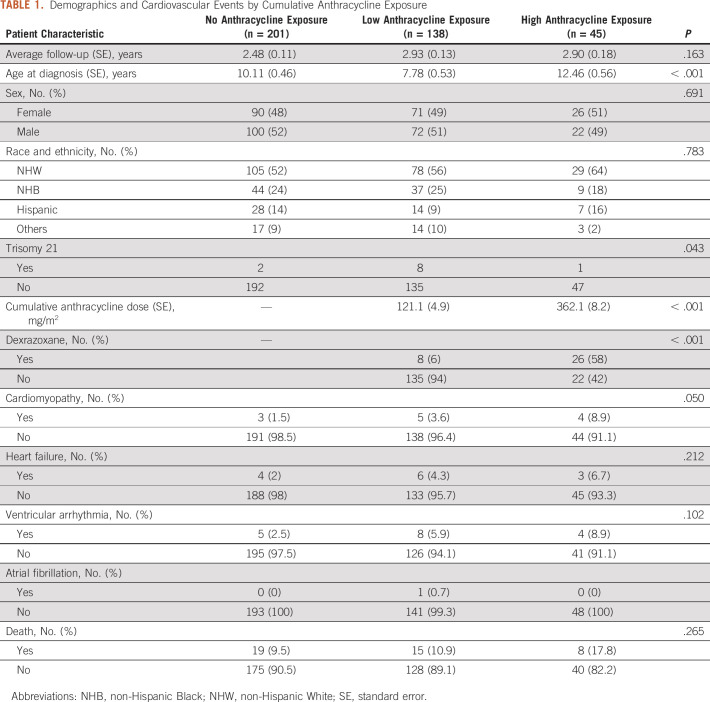
Demographics and Cardiovascular Events by Cumulative Anthracycline Exposure

### Quality Assurance of SQL Coding for Anthracycline Exposure

Manual EHR review of anthracycline exposures was conducted for quality assurance and was highly consistent with the automatic extraction through SQL (Table 2). Of the 10% reviewed (n = 19), one patient with confirmed daunorubicin administration did not have a documented body surface area, which resulted in an underestimate of the cumulative anthracycline exposure by 23 mg/m^2^. Additionally, we manually reviewed 10% of patients in the cancer registry that did not have anthracycline exposure from SQL automatic extraction (n = 53). Of these patients, 85% (n = 46) had no record of anthracycline administration, 13% (n = 7) were pediatric hematopoietic stem cell transplant (HSCT) patients with documented anthracycline exposure at an outside institution, and one was a medical oncology patient who received anthracyclines at an outside institution.

**TABLE 2.**
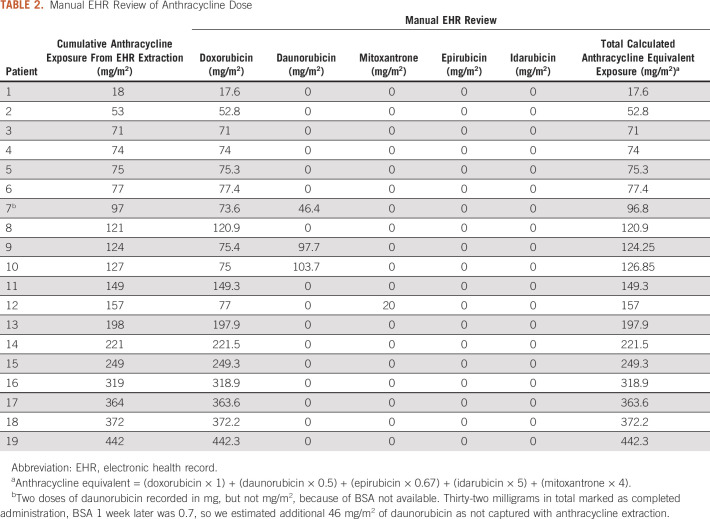
Manual EHR Review of Anthracycline Dose

### Cardiac Events Among Anthracycline-Exposed Groups

DEDUCE facilitated the automatic extraction of all ICD-9-CM and ICD-10-CM codes from the EHR and the identification of diagnoses for cardiovascular outcomes of interest (Table 1). Of patients with high anthracycline exposure, 8.9% had documented cardiomyopathy, 6.7% had heart failure, and 8.9% had ventricular arrhythmia during or after therapy. There were no significant differences in cardiomyopathy (*P* = .050), heart failure (*P* = .212), ventricular arrhythmia (*P* = .102), or death (*P* = .265) among the anthracycline exposure groups (Table 1) during the follow-up period. Diagnoses of CVD before the date of initial diagnosis for malignancy were excluded.

### Sensitivity Analysis for ICD-9-CM and ICD-10-CM Classification and Manual Chart Review of Echocardiogram for Anthracycline-Exposed Groups

All echocardiogram reports from the anthracycline-exposed groups were manually reviewed. Excluding patients with a previous diagnosis of heart failure, 4.3% of patients met parameters for heart failure (n = 8) and 4.8% of patients had an ICD-9-CM and ICD-10-CM code for heart failure (n = 9). This yielded a sensitivity of 75% and a specificity of 98% of this ICD-9-CM and ICD-10-CM coding approach to detect heart failure parameters on echocardiogram with a positive predictive value of 67% and a negative predictive value of 99%. Refinement of the ICD-based coding schema to include cardiac arrest increased the sensitivity to 87.5% and decreased the positive predictive value to 58% (Table 3). A manual chart review of all discordant cases was performed (Appendix Tables A4 and A5).

**TABLE 3.**
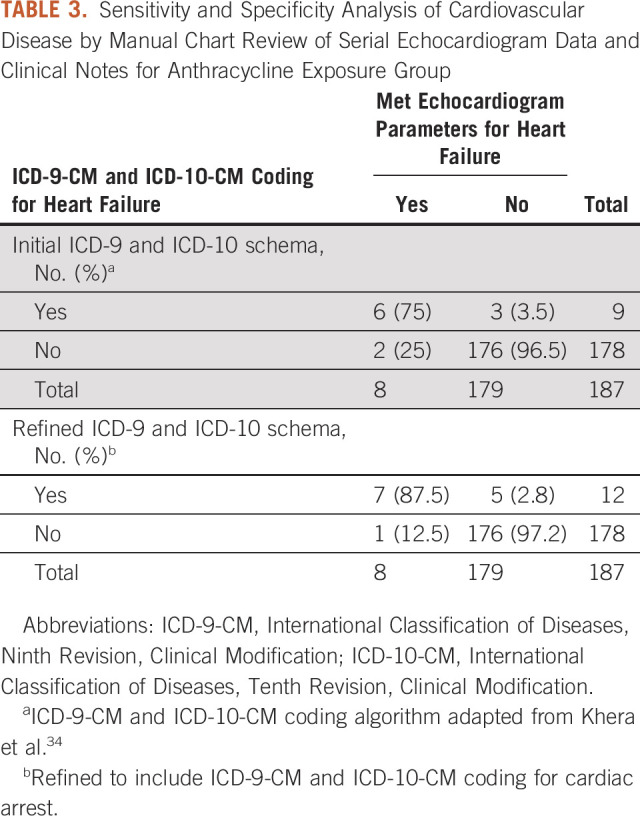
Sensitivity and Specificity Analysis of Cardiovascular Disease by Manual Chart Review of Serial Echocardiogram Data and Clinical Notes for Anthracycline Exposure Group

### Guideline Adherence to Echocardiogram Surveillance Recommendations

Within the anthracycline-exposed cohorts, we performed a landmark analysis to ensure equivalent follow-up time to assess adherence to guideline recommendations for surveillance echocardiograms. For the group recommended for echocardiogram surveillance every 2 years, we excluded patients with last anthracycline administration after December 2019 (n = 1) and death during the follow-up period (n = 8) to allow for a 27-month follow-up time. For the group recommended to receive echocardiogram surveillance every 5 years, we excluded patients with last anthracycline administration after December 2016 (n = 88) and death during the follow-up period (n = 15) to allow for a 63-month follow-up time. Twenty-two percent of patients were not adherent to the COG Long-Term Follow-Up Guidelines for echocardiogram surveillance (n = 17; Table 4). Of these 17 patients, three did not retrieve any CPT code for echocardiogram on automatic data extraction. With manual chart review, one met criteria for guideline adherence, one did not meet criteria for guideline adherence, and one was treated exclusively by medical oncology and did not complete the recommended treatment. There were no differences among the anthracycline exposure groups (*P* = .666), age at diagnosis (*P* = .601), and sex (*P* = .983).

**TABLE 4.**
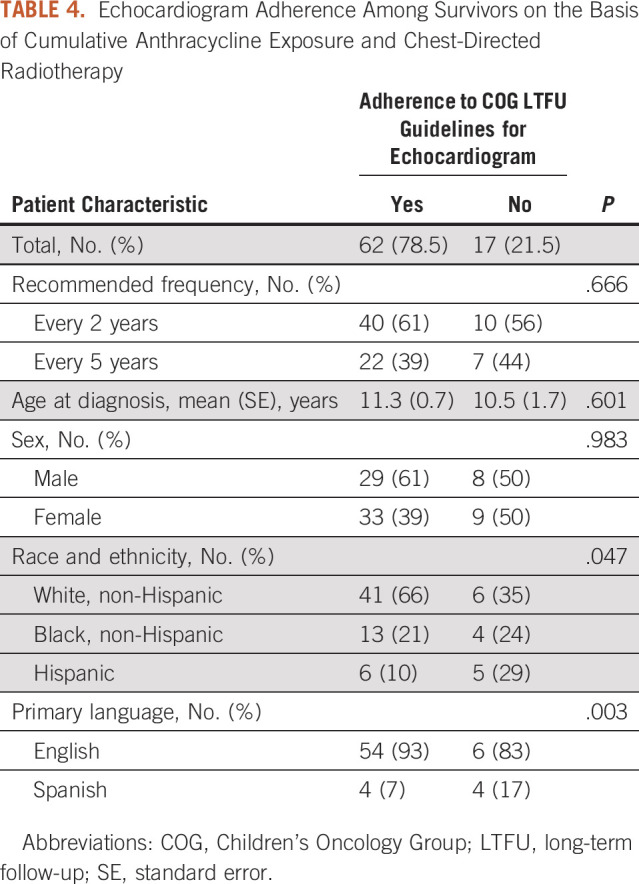
Echocardiogram Adherence Among Survivors on the Basis of Cumulative Anthracycline Exposure and Chest-Directed Radiotherapy

### Disparities in Echocardiogram Surveillance by Race and Ethnicity and Primary Language

On the basis of χ^2^ tests, there was a significant association between race and ethnicity (*P* = .047) and primary language (*P* = .003) and adherence to echocardiogram surveillance guidelines. Eight-seven percent of non-Hispanic White patients received echocardiograms within the recommended time period compared with 76% of non-Hispanic Black patients and 55% of Hispanic patients. With regard to primary language, 90% of English-speaking patients received guideline-based care for echocardiogram surveillance compared with 50% of Spanish-speaking patients (Table 4).

## DISCUSSION

This study supports the utility of SQL to automatically extract cumulative anthracycline exposure from the EHR, using cancer registry data as a base cohort. Manual EHR review revealed excellent concordance of 95% with results from the automated SQL data extraction. One patient had two missed doses of daunorubicin because of lack of a recorded body surface area at the time of administration; however, this did not lead to misclassification for recommended echocardiogram frequency. The discrete structure of the lifetime dose tracking of anthracycline exposure in Epic, accessible through SQL, represents a model for other key chemotherapy exposures to monitor the late effects.^41,42^

The major limitations for these automated approaches for EHR data extraction include the potential to underestimate exposure to anthracyclines because of the receipt of some care at outside institutions. The exclusion of nonanalytic cases helps mitigate undocumented anthracycline exposures. Nevertheless, 15% of patients had free text documentation of anthracycline administration at outside institutions. Nearly all of these patients were pediatric hematopoietic stem-cell transplant patients who received a portion of their care at an outside institution. This led to misclassification of patients as having no anthracycline exposure when they likely had low anthracycline exposure. Anthracycline exposure is a critical data element, for both care during active treatment and survivorship, to inform the risk of cardiotoxicity. This underscores the need for increased interoperability of these structured data elements unique to oncology to facilitate health information exchange for treatment received at outside institutions.

The application of established ICD-9-CM and ICD-10-CM coding schema for heart failure^33-35^ illustrates the opportunity to capture early CVD for children with cancer. The use of dexrazoxane for cardioprotection may account for the lack of significant difference in cardiac events between the low and high anthracycline groups; however, this may also reflect insufficient power because of the small sample size. Interinstitutional collaborations for surveillance of cardiac events would increase sample size and benefit from the informatics methods presented in this study. Manual review of echocardiogram parameters yielded a modest sensitivity of 75% and a positive predictive value of 67%, which calls for further refinement to detect heart failure on a population health level. Current advances in natural language processing^43-45^ and machine learning^46^ offer next steps to improve the detection of heart failure in the general population.

Echocardiogram adherence,^38^ on the basis of CPT coding and enhanced by manual EHR review for quality assurance, showed that nearly a fifth of survivors with anthracycline exposure received suboptimal guideline-based care during the early follow-up period. With regard to echocardiogram adherence, this single institution does not capture echocardiograms at outside facilities, as an absence of data does not definitively equate to an absence of care. Nevertheless, the tight follow-up window after the last known anthracycline administration represents a relatively early period when patients would more likely be seen for active follow-up at the primary treatment site. The differences in guideline-based echocardiogram surveillance by race and ethnicity and primary language raise questions of health equity. Although adjustment for sociodemographic or language barriers was limited by cohort size, this observation compels further investigation to the root causes of these disparities to promote equitable survivorship-focused care.

Knowledge gaps for interventions to improve guideline-adherent care for survivors^47^ highlight the need for clinical informatics approaches, such as SQL and ICD or CPT coding, to facilitate population health–level solutions. The significant heterogeneity in treatment exposures and risk of late effects^48-51^ underscores the complexity of providing appropriate care for survivors. Real-world data from the EHR serve as scaffolding for future implementation science-based interventions across the oncology continuum, including survivorship,^52^ through the iterative, evidence-generating dynamic sustainability framework^53^ to refine clinical practice. While longitudinal cohort studies, such as CCSS,^54^ offer considerable insight, future advances in survivorship care would benefit from a learning health systems approach given the dynamic nature of pediatric oncology care with recent de-escalation of treatment regimens, newer targeted agents, cardioprotective strategies, and immunotherapy. For cardiomyopathy specifically, review of existing guidelines calls for ongoing evaluation of potential interventions to mitigate late effects.^15^ Subspecialty clinic attendance significantly improves adherence to cardiomyopathy screening guidelines for adult survivors.^55^

The structured data elements presented in our analysis for anthracycline exposures and CVD among children with cancer align well with national efforts to advance clinical research informatics. The mCODE initiative seeks to enhance cancer care delivery through conformance to Fast Healthcare Interoperability Resources (FHIR) standards, approved by HL7, to increase data interoperability.^28^ Using SMART on FHIR provides a level of EHR independence.^56^ Although the work presented in this study was done using Epic, it should be replicable with any EHR vendor-supporting FHIR. Applications in precision medicine in oncology for clinical decision support^57^ and recent advances with the SMART/HL7 bulk data access standard^58^ bolster future directions for population health in pediatric oncology. The integration of cancer registry and EHR data provides a useful framework, albeit the main limitations in our study being the relatively small sample size and lack of robust follow-up time to detect late effects. Longitudinal, interinstitutional collaborations that leverage structured data elements and interoperability within pediatric oncology will be increasingly important as new agents with potential cardiotoxicity change the landscape of survivorship care in the future.^59,60^

## Figures and Tables

**TABLE A1.**
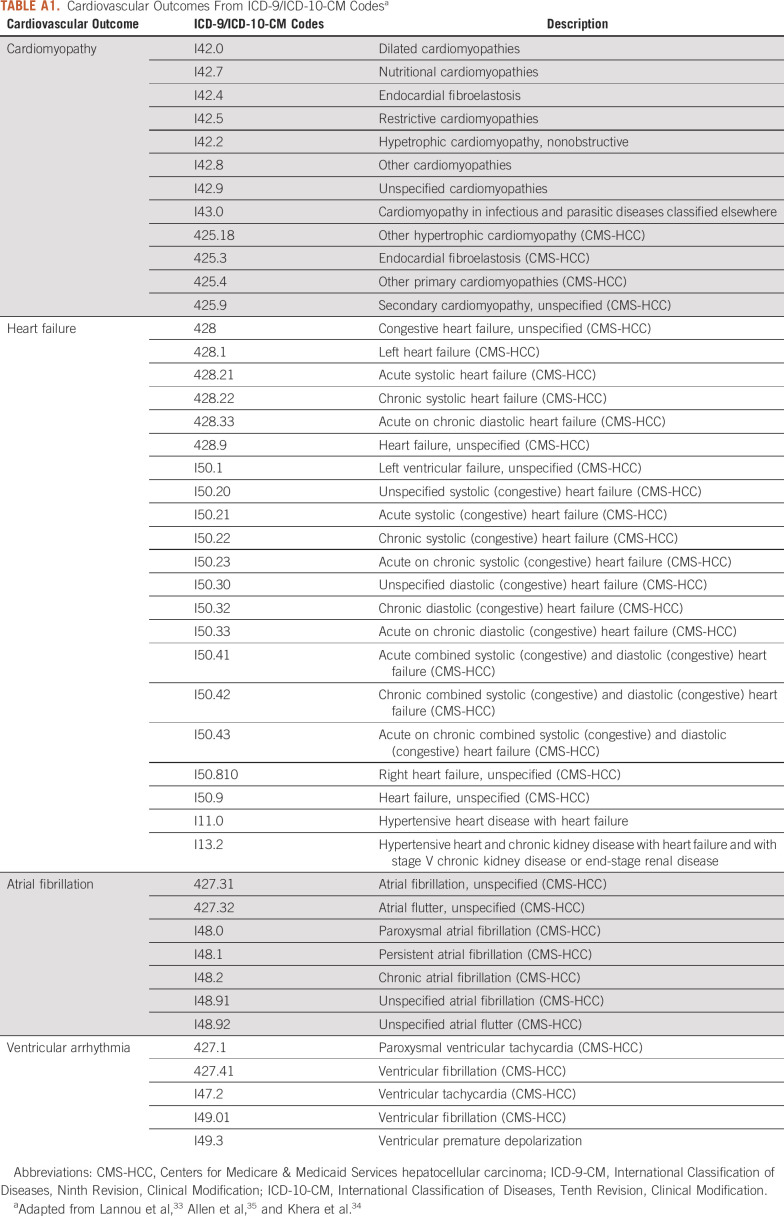
Cardiovascular Outcomes From ICD-9/ICD-10-CM Codes^a^

**TABLE A2.**
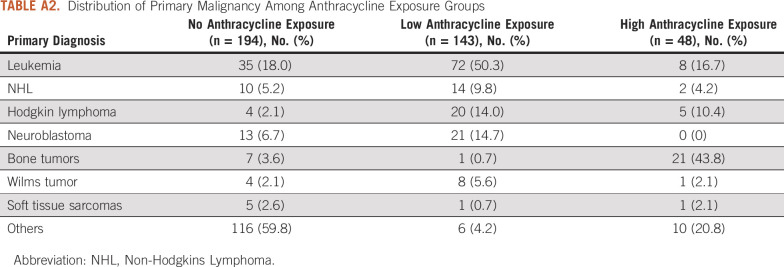
Distribution of Primary Malignancy Among Anthracycline Exposure Groups

**TABLE A3.**
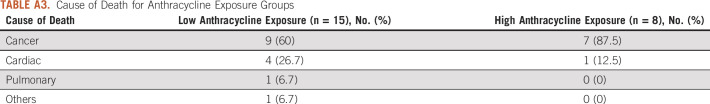
Cause of Death for Anthracycline Exposure Groups

**TABLE A4.**
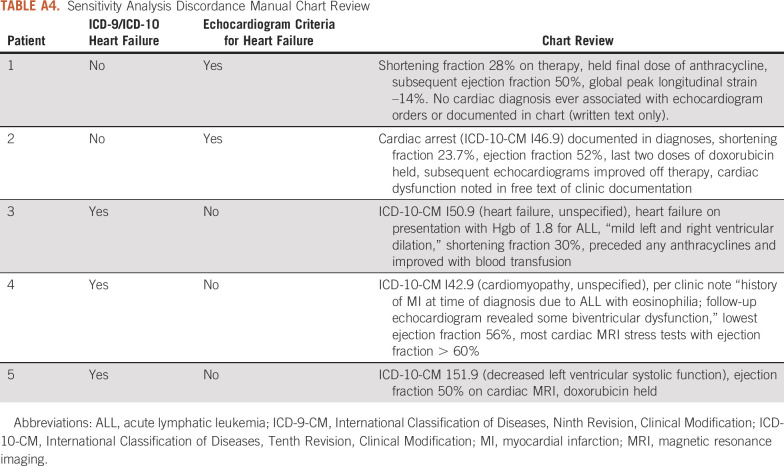
Sensitivity Analysis Discordance Manual Chart Review

**TABLE A5.**
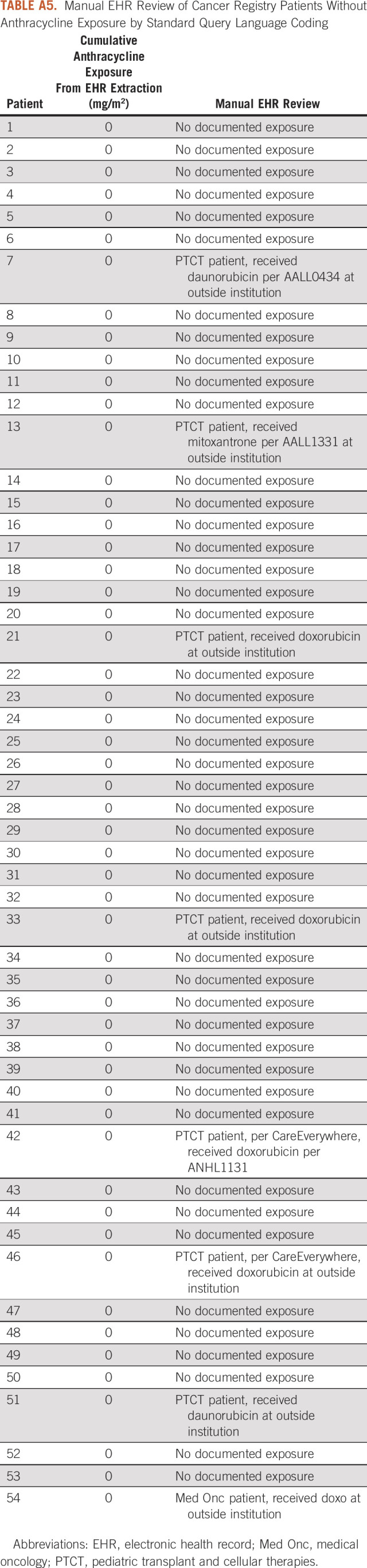
Manual EHR Review of Cancer Registry Patients Without Anthracycline Exposure by Standard Query Language Coding

## Data Availability

The data that support the findings of this study are available on request from the corresponding author. The data are not publicly available due to privacy or ethical restrictions. While the SQL is not allowed to be published within the manuscript due to contractual obligations to Epic, authors can request that the query be published within Epic's query library that is accessible to all Epic users (including researchers).
